# Risk of type 2 diabetes and *KCNJ11* gene polymorphisms: a nested case–control study and meta-analysis

**DOI:** 10.1038/s41598-022-24931-x

**Published:** 2022-12-01

**Authors:** Maryam Moazzam-Jazi, Leila Najd-Hassan-Bonab, Sajedeh Masjoudi, Maryam Tohidi, Mehdi Hedayati, Fereidoun Azizi, Maryam S. Daneshpour

**Affiliations:** 1 Cellular, and Molecular Endocrine Research Center, Research Institute for Endocrine Sciences, Shahid Beheshti University of Medical Sciences, Tehran, Iran; 2 Prevention of Metabolic Disorder Research Center, Research Institute for Endocrine Sciences, Shahid Beheshti University of Medical Sciences, Tehran, Iran; 3 Endocrine Research Center, Research Institute for Endocrine Sciences, Shahid Beheshti University of Medical Sciences, Tehran, Iran

**Keywords:** Genetics, Microbiology, Biomarkers, Diseases, Endocrinology, Medical research

## Abstract

Due to the central role in insulin secretion, the potassium inwardly-rectifying channel subfamily J member 11 (*KCNJ11*) gene is one of the essential genes for type 2 diabetes (T2D) predisposition. However, the relevance of this gene to T2D development is not consistent among diverse populations. In the current study, we aim to capture the possible association of common *KCNJ11* variants across Iranian adults, followed by a meta-analysis. We found that the tested variants of *KCNJ11* have not contributed to T2D incidence in Iranian adults, consistent with similar insulin secretion levels among individuals with different genotypes. The integration of our results with 72 eligible published case–control studies (41,372 cases and 47,570 controls) as a meta-analysis demonstrated rs5219 and rs5215 are significantly associated with the increased T2D susceptibility under different genetic models. Nevertheless, the stratified analysis according to ethnicity showed rs5219 is involved in the T2D risk among disparate populations, including American, East Asian, European, and Greater Middle Eastern, but not South Asian. Additionally, the meta-regression analysis demonstrated that the sample size of both case and control groups was significantly associated with the magnitude of pooled genetic effect size. The present study can expand our knowledge about the *KCNJ11* common variant's contributions to T2D incidence, which is valuable for designing SNP-based panels for potential clinical applications in precision medicine. It also highlights the importance of similar sample sizes for avoiding high heterogeneity and conducting a more precise meta-analysis.

## Introduction

Type 2 diabetes (T2D), the most common form of diabetes, is one of the leading life-threatening diseases worldwide, with 4.2 million deaths globally, as reported in 2019 by International Diabetes Federation (IDF) consortium^1^. In recent years, urbanization and a sedentary lifestyle have been significantly increasing the T2D incidence. It is estimated the number of affected individuals with this metabolic disorder may enhance up to about 642 million by 2040^2^.

Type 2 diabetes is raised by genetic, non-genetic (environmental) factors, and interaction between them^3,4^. The genetic architecture of the disease is driven by multiple causal genetic variants with small to modest effect sizes, which influence either sufficient insulin secretion by pancreatic beta cells or proper insulin response^5^. Genome-wide association studies have identified many genetic variants associated with T2D and fasting glucose levels^6^. In recent years, several studies revealed the association of different genetic variants with the risk of T2D development among Iranians^7,8^. Sadeghi et al. (2021) deciphered the positive association of rs28514894 and rs2303044 belonging to the *NR1H2* gene with the risk of T2D development. They also showed a significant difference in the blood urea nitrogen levels among diabetic carriers of these polymorphisms^9^. It has been reported that polymorphisms at the 3’UTR of *SLC30A8* (rs2466293 and rs2466294) probably increased the Iranian susceptibility to T2D by affecting the binding site of some miRNAs and reducing the stability of *SLC30A8* mRNA transcripts^10^. In addition to the coding genes, genetic variants on the non-coding genes contribute to type 2 diabetes development, pointing to the crucial role of gene expression regulation in the disease incidence. According to Jahantigh et al., some polymorphisms at the promoter region of the miR-143/145 cluster could significantly influence the T2D development in the Iranian population. The involvement of target genes of these miRNAs in the glucose and lipid metabolism pathways may explain their contribution to the T2D predisposition^11^. *HOTAIR*, one of the well-documented long non-coding RNAs, has a prominent role in glucose metabolism regulation. Sargazi et al. figured out that several SNPs belonging to this gene can affect the risk of type 2 development through modulating various biological pathways^12^. Among the numerous T2D-related genes, the adenosine triphosphate-sensitive potassium channel (*KATP*) plays a major role in regulating glucose-stimulated insulin secretion by beta cells through coupling cell membrane potential with cell metabolism. The KATP is a homo-tetramer of potassium inward channel (Kir6.2) and regulatory sulfonylurea receptor SUR1 subunits (ABCC8). The Kir6.2 subunit is coded by potassium inwardly-rectifying channel subfamily J member 11 (*KCNJ11*) gene with high expression in the pancreas. Mutation in both genes, *KCNJ11* and *ABCC8*, can result in neonatal diabetes and congenital hyperinsulinemia in humans^4,5^. In 2002, Schwanstecher et al. reported that a non-synonymous polymorphism in *KCNJ11* (rs5219) that substitutes glutamate for lysine at position 23 (E23K). It could change protein function via inducing pancreatic beta-cell over-activity, leading to defective insulin secretion^15^. A recent simulation study by computational approaches demonstrated the normal coupling between the open and close states of the KATP channel, resulting in normal insulin secretion, unlike the mutant Kir6.2^16^. Hence, this polymorphism plays a key role in developing T2D as also represented by various association studies in different populations^8–10^. However, the effect of rs5219 on T2D susceptibility is not consistent among diverse ethnicities. For instance, the lack of association of this polymorphism with T2D was reported in Iranian^19^, Emirati^20^, Moroccan^21^, and Asian Indian^13^ populations. rs5210 and rs5215 are other *KCNJ11* well-known polymorphisms. The rs5210 and rs5215 are located at the 3’ untranslated region (UTR) and the coding region of the *KCNJ11* gene, respectively, which the later polymorphism alters the amino acid of valine to isoleucine at residue 250 of the corresponding protein^13^. Although researchers have less surveyed these polymorphisms of *KCNJ11*, their inconsistent contribution to T2D development was also reported across populations. In 2007, Koo et al. claimed that both rs5210 and rs5215 were significantly associated with type 2 diabetes incidence in the Korean population^22^, but they have not been confirmed in the Mexican population^23^. Due to the potentially broad clinical applications of single genetic polymorphisms in precision medicine for early diagnosis and treatment, it is critical to recognize their explicit relevance to disease incidence. However, different sample sizes and ethnic groups used by various researchers limited our ability to realize the clear effect of the genetic polymorphisms on complex disease development, such as type 2 diabetes. A meta-analysis of the genetic association studies is an efficient approach to gain a better understanding of genetic variants' impact on the disease incidence. Despite the importance of *KCNJ11* polymorphisms, the lack of a comprehensive genetic association study among Iranians, a population with an unknown genetic makeup, motivated us to discover the possible involvement of the *KCNJ11* polymorphisms in type 2 diabetes development across Iranians. The current study aims to (1) survey the association of common *KCNJ11* polymorphisms (rs5210, rs5215, and rs5219) with T2D risk in a large sample size of the Iranian population; (2) examine pancreatic-cell function in individuals with different rs5219 genotypes; and (3) integrate our findings with all previous case–control studies and present the most comprehensive and up-to-date meta-analysis to precisely uncover the impact of common *KCNJ11* polymorphisms on type 2 diabetes predisposition; (4) identify the probable sources of heterogeneity among studies used for the meta-analysis.

## Results

### Nested case–control study

#### Population description

The nested case–control study was performed on 1326 diabetic and 1594 non-diabetic unrelated adults who participated in the ongoing TCGS cohort project; the average age of the case and control groups were 50.41 and 37.5 years, respectively. Demographic and biochemical characteristics of participants at the baseline are available in Supplementary Table S1. The power of our analysis was 83% with the above sample size.

#### Association of *KCNJ11* with type 2 diabetes

The genotype distribution of three well-known *KCNJ11* SNPs, rs5210, rs5215, and rs5219 complied with the Hardy–Weinberg equilibrium (p-value > 0.05). According to our association results under six genetic models (recessive, dominant, additive, over-dominant, codominant heterozygous, and codominant homozygous), none of the *KCNJ11* SNPs could significantly influence T2D development in Iranian adults. The same finding was obtained in the model adjusted for age, sex, and BMI as covariates (Supplementary Table S2).

#### Pancreatic β-cell function among participants with different rs5219 genotypes

We surveyed the insulin secretion level by estimating β-cell function and comparing it among non-diabetic individuals with zero, one, and two risk alleles of rs5219. The current analysis was done on 808 participants of interest whose fasting insulin data was available. Our results indicated that the difference in insulin secretion was not statistically significant among various rs5219 genotypes (p-value = 0.69, F (2,805) = 0.37) (Supplementary Table S3), proposing the lack of involvement of this polymorphism in altered insulin secretion.

### Meta-analysis study

#### Characteristics of eligible studies

**Figure 1:**
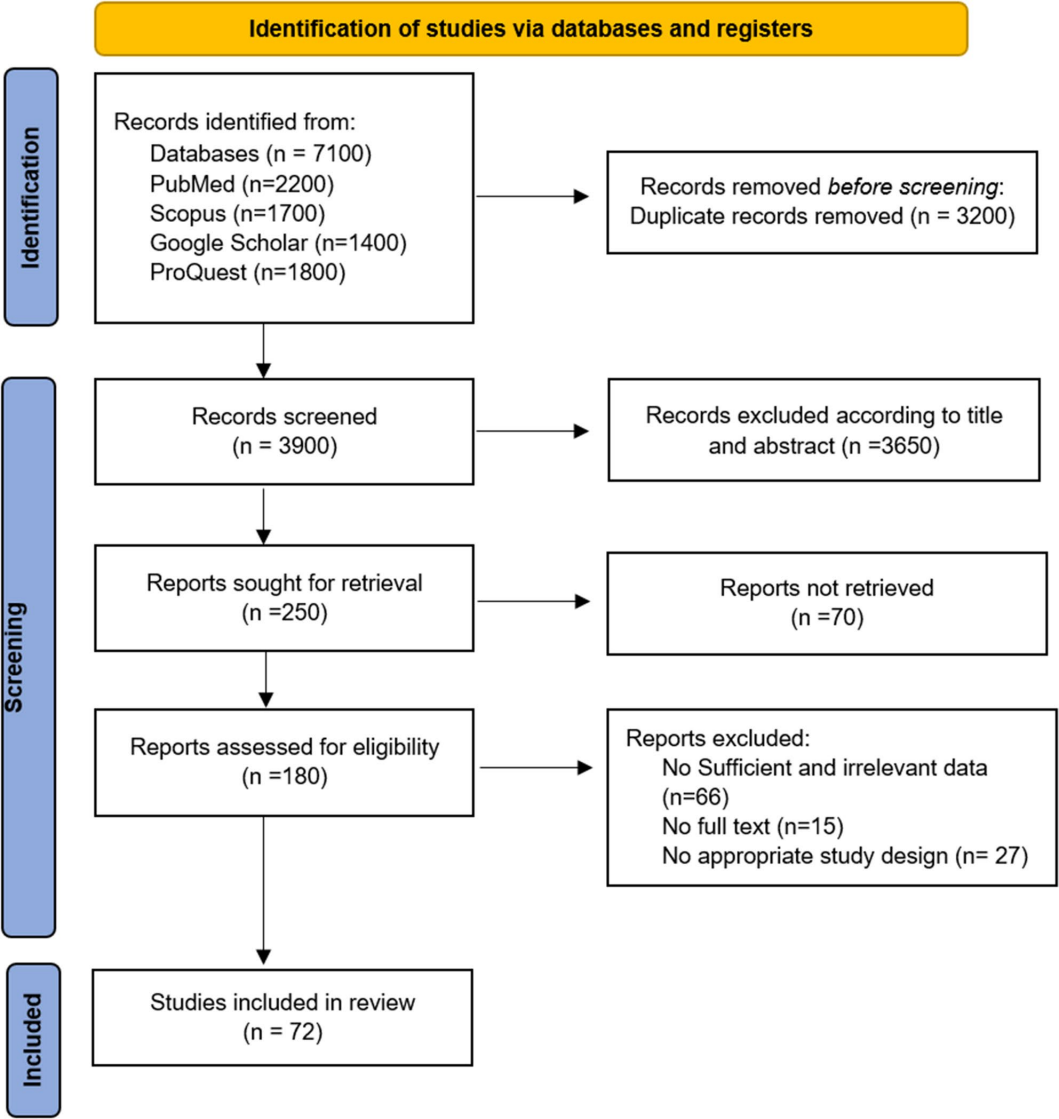
Preferred reporting items for systematic reviews and meta-analyses (PRISMA) flow chart for study selection process.

We obtained 7100 articles from our comprehensive initial literature search. After excluding the improper and duplicate papers, 72 articles were found to be qualified for meta-analysis. The study selection process is illustrated in Fig. 1. A total of 41,372 cases and 47,570 controls from Africa, America, Europe, Eastern Asia, Southern Asia, and Greater Middle Eastern were covered with the 72 included case–control studies. The main features of the eligible studies for the present meta-analysis are summarized in Table 1.

**Table 1:** Characteristics of the studies included in the present meta-analysis (n = 72).

First author et al	Publication year	Population	Ancestry category	Genotyping method	Sample size case	Sample size control	p-value HWE	References
**rs5219**								
Sakura et al	1996	United Kingdom	European	PCR-SSCP	100	82	0.365	^49^
Inoue et al	1997	United Kingdom	European	PCR-SSCP	172	95	0.1466	^50^
Hani et al	1998	France	European	PCR-SSCP	191	114	0.9689	^51^
Gloyn et al	2003	United Kingdom	European	PCR-SSCP	854	1182	0.7025	^52^
Nielsen et al	2003	Denmark	European	PCR	803	862	0.9434	^53^
Dam et al	2005	Netherland	European	PCR–RFLP	192	296	0.7083	^54^
Hansen et al	2005	Denmark	European	PCR–RFLP	1187	1454	0.7025	^24^
Yokoi et al	2006	Japanese	East Asian	Mass array	1590	1244	0.7545	^55^
Cejková et al	2007	Czech	European	PCR–RFLP	172	113	0.5324	^56^
Qi et al	2007	USA	American	TaqMan	682	1078	0.6614	^57^
Sakamoto et al	2007	Japanese	East Asian	TaqMan	906	889	0.82	^58^
Koo et al	2007	Korean	East Asian	TaqMan	758	630	0.2236	^22^
Doi et al	2007	Japanese	East Asian	TaqMan	550	1433	0.73	^59^
Sanghera et al	2008	Asian Indian Sikhs	South Asian	TaqMan	532	374	0.6824	^13^
Willer et al	2008	France	European	Mass array	287	2684	0.7891	^60^
Alsmadi et al	2008	Saudi Arab	Greater Middle East	TaqMan	550	335	0.6614	^61^
Chistiakov et al	2008	Russian	Other	PCR–RFLP	129	117	0.0716	^62^
Cauchi et al	2008	French	European	TaqMan	2734	4234	0.79	^63^
Thorsby et al	2009	Norway	European	PCR–RFLP	750	1879	0.4551	^64^
Cornelis et al	2009	USA	American	OpenArray SNP Genotyping	2709	3344	0.9804	^65^
Zhou et al	2009	Chinese	East Asian	TaqMan	1848	1910	0.0716	^66^
Ezzidi et al	2009	Tunisia	Greater Middle East	TaqMan	805	521	0.4551	^67^
Wang et al	2009	Chinese	East Asian	SNaPshot multiplex system	396	387	0.7	^68^
Tabara et al	2009	Japanese	East Asian	TaqMan	484	397	0.65	^69^
Yu et al	2010	Chinese	East Asian	PCR–RFLP	295	188	0.73	^70^
Wen et al	2010	Chinese	East Asian	Mass array	1165	1135	0.3363	^71^
Neuman et al	2010	Ashkenazi Jewish	Other	Pyrosequencing	573	843	0.5146	^72^
Boodram et al	2011	Indo-Trinidadians	North American	PCR	168	61	0.2348	^73^
Cheung et al	2011	Hong Kong	East Asian	TaqMan	198	1185	0.6614	^74^
Gonen et al	2012	Turkish	Greater Middle East	PCR-SSCP	162	79	0.0017	^75^
lwata et al	2012	Japanese	East Asian	TaqMan SNP Genotyping	724	763	0.2348	^76^
Abdelhamid et al	2013	Mauritanian	African	pcr	135	135	0.2723	^77^
Benrahma et al	2014	Moroccan	Greater Middle East	TaqMan	248	248	0.7025	^21^
Keshavarz et al	2014	Iranian	Greater Middle East	TaqMan	400	420	0.9434	^19^
Lasram et al	2014	Tunisians and Arabs	Greater Middle East	TaqMan	250	267	0.7025	^78^
Phani et al	2014	South Indian	South Asian	TETRA-ARMS	399	400	0.379	^79^
Sokolova et al	2015	Russian	Other	TaqMan	1384	414	0.5324	^33^
Zhuang et al	2015	Chinese	East Asian	PCR-direct sequencing	175	182	0.8395	^80^
Rastegari et al	2015	Iranian	Greater Middle East	PCR–RFLP	20	20	0.0533	^81^
Qian et al	2015	Chinese Han	East Asian	TaqMan	1192	1192	0.7025	^82^
Nikitin et al	2015	Russian	Other	Real-time PCR	440	265	0.36	^17^
Nikitin et al	2017	Russian	Other	TaqMan	862	443	0.0533	^17^
Souza et al	2017	Euro-Brazilian	South American	PCR–RFLP	141	217	0.5146	^83^
Rizvi et al	2018	Indian	South Asian	PCR–RFLP	200	200	0.0065	^84^
Engwa et al	2018	Nigerian	African	PCR–RFLP	73	75	0.01	^85^
Makhzoom et al	2019	Syria	Greater Middle East	PCR–RFLP	75	63	0.0104	^86^
Sarkar et al	2019	North East Indian	South Asian	PCR–RFLP	155	100	0.7545	^87^
Ali et al	2019	Emirati	Greater Middle East	TaqMan	153	264	0.5324	^20^
Muftin et al	2019	Iraqian	Greater Middle East	PCR–RFLP	40	20	0.6776	^88^
Isakova et al	2019	Kyrgyz	Other	PCR–RFLP	114	109	0.3865	^89^
Aswathi et al	2020	South Indian	South Asian	ARMS-PCR	218	214	0.4551	^90^
li et al	2020	Chinese	East Asian	Mass array	1194	1292	0.5793	^91^
Moazzam-Jazi et al	2022	Iranian	Greater Middle East	Illumina chip	1321	1596	0.8293	Present study
**rs5215**								
Inoue et al	1997	Caucasian	European	PCR-SSCP	203	96	0.002	^50^
Hani et al	1998	France	European	PCR-SSCP	187	113	0.8509	^51^
Koo et al	2007	Korean	East Asian	TaqMan	761	611	0.078	^22^
Boodram et al	2011	Indo-Trinidadians	North American	sequencing	66	59	0.8509	^73^
Gonen et al	2012	Turkish	Greater Middle East	PCR-SSCP	133	112	0.8509	^75^
Phani et al	2014	South Indian	South Asian	TETRA-ARMS	400	400	0.8509	^79^
Qian et al	2015	Chinese Han	East Asian	TaqMan	1192	1185	0.8509	^82^
Sikhayeva et al	2017	Kazakh	East Asian	TaqMan	375	829	0.9662	^92^
Althwanay et al	2020	Saudi Arab	Greater Middle East	TaqMan	49	39	0.6177	^93^
Moazzam-Jazi et al	2022	Iranian	South Asian	Illumina chip	1324	1595	0.9662	Present study
**rs5210**								
Koo et al	2007	Korean	East Asian	TaqMan	758	163	0.0792	^22^
Sakamoto et al	2007	Japanese	East Asian	TaqMan	897	188	0.7671	^58^
Khan et al	2015	India	South Asian	PCR–RFLP	250	136	0.0045	^94^
Khan et al	2019	India	South Asian	PCR–RFLP	300	58	0.1383	^95^
Khan et al	2020	India	South Asian	PCR–RFLP	300	50	0.1383	^96^
Yiping et al	2020	Chinese	East Asian	Mass array	1194	368	0.5794	^91^
Malekizadeh et al	2021	Iranian	Greater Middle East	Sanger sequencing	111	5	0.5794	^97^
Moazzam-Jazi et al	2022	Iranian	Greater Middle East	Illumina chip	1287	777	0.1215	Present study
Alqadri	2022	Saudi Arab	Greater Middle East	PCR–RFLP	102	13	0.0225	^89^

#### Association meta-analysis of *KCNJ11* polymorphisms with type 2 diabetes

##### rs5219

**Figure 2:**
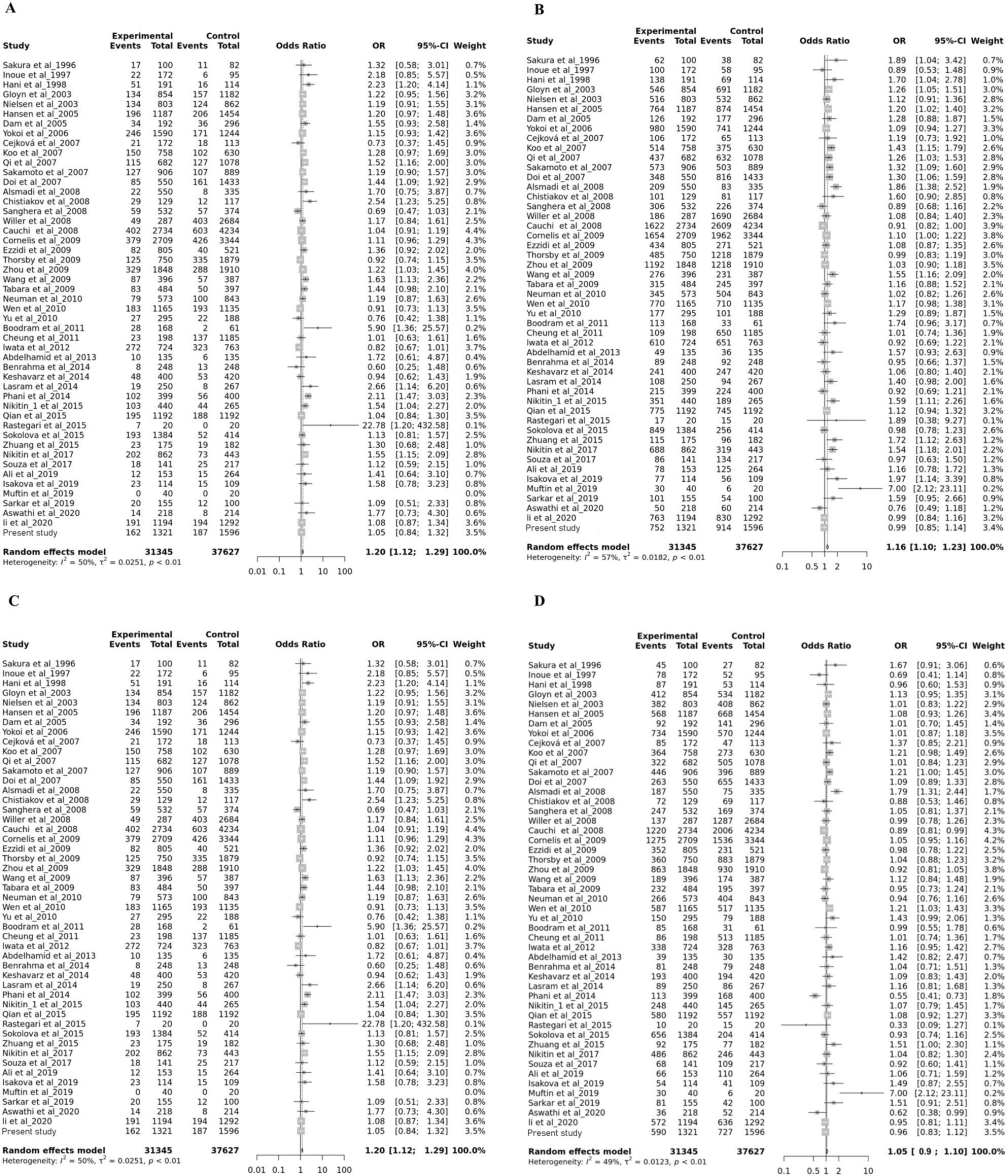
Forest plot for the association between rs5219 and type 2 diabetes risk under different genetic models, allele contrast (**A**), recessive (**B**), dominant (**C**), and over-dominant (**D**). Diamond shows the pooled odds ratio size and its 95% CI.

There were 53 eligible studies for the rs5219 polymorphism; four studies were excluded due to deviation from the Hardy–Weinberg equilibrium. A total of 49 studies composed of 31,345 cases and 37,627 controls were used for the meta-analysis (Table 1). In the overall analysis, we detected the significant high heterogeneity with I^2^ value of 49–61% among the included studies (p-value < 0.05) under all four genetic models. Therefore, the random-effects model was applied for the analysis, representing a significant positive association of rs5219 with type 2 diabetes development under all genetic models except for the over-dominant model (Fig. 2). The largest effect size (OR) of this polymorphism was achieved under recessive model (combined OR = 1.2, CI 1.12–1.29, p-value = 5.51E−07) (Table 2). Considering the cutoff value of 0.2 suggested by the FPRP test developer, the calculated FPRP value was less the specified threshold for all the significant associations, confirming our obtained positive results. However, the stratified analysis according to ethnicity was returned slightly different results for various populations. Here, based on the p-value cutoff of 0.05 for the heterogeneity test, we opted for the random-effects or the fixed-effect model in each population. Interestingly, rs5219 is significantly associated with T2D incidence in all ethnicities under at least one genetic model, except for Southern Asian where we detected no association under any genetic models. However, the FPRP value obtained for the Greater Middle Eastern ancestry was 0.5 (Table 3). Since the ethnic groups of African and Central Asian encompassed only one study, these groups were not considered for the stratified analysis.

**Table 2:** Association meta-analysis of *KCNJ11* polymorphisms in the general population.

SNP	Allele contrast					Recessive					Dominant					Over-dominant			
	Sample size (case/control)	OR (95% CI)	p-value^1^	p-value^2^	FPRP	Sample size (case/control)	OR (95% CI)	p-value^1^	p-value^2^	FPRP	Sample size (case/control)	OR (95% CI)	p-value^1^	p-value^2^	FPRP	Sample size (case/control)	OR (95% CI)	p-value^1^	p-value^2^
rs5210	9694/9284	1.04 (0.9–1.23)	0.6	0.268	–	4847/4642	0.92 (0.7–1.14)	0.438	0.434	–	4847/4642	1.12 (0.9–1.41)	0.345	0.387	–	4847/4642	1.12 (0.99–1.27)	0.074	0.185
rs5215	8974/9886	1.5 (0.87–2.5)	0.14	0.85	–	4487/4943	2.07 (1.17–3.6)	0.013	0.224	0.24	4487/4943	1.41 (0.7–2.83)	0.33	0.756	–	4487/4943	1.17 (0.7–1.98)	0.54	0.69
rs5219	6260/7524	1.14 (1.09–1.19)	9.00E−10	1.00E−04	< .0001	31,345/37,627	1.2 (1.12–1.29)	5.51E−07	3.80E−03	< .0001	31,345/37,627	1.16 (1.1–1.23)	8.29E−08	2.00E−04	< .0001	31,345/37,627	1.05 (0.9–1.1)	0.07	0.091

The FPRP analysis was done for the significant associations. The FPRP level of noteworthiness is 0.2

*OR* odds ratio, *CI* confidence interval, *FPRP* false-positive report probability.

^1^p-value of association test.

^2^p-value of Egger’s test.

**Table 3:** Association meta-analysis of rs5219 in the subgroup analysis.

Ancestry	Allele contrast				Recessive				Dominant				Over-dominant			
	Sample size (case/control)	OR (95% CI)	p-value	FPRP	Sample size (case/control)	OR (95% CI)	p-value	FPRP	Sample size (case/control)	OR (95% CI)	p-value	FPRP	Sample size (case/control)	OR (95% CI)	p-value	FPRP
American	7400/9400	1.17 (1.01–1.26)	3.37E−02	1E−03	3700/4700	1.33 (0.97–1.8)	0.07	–	3700/4700	1.13 (1.04–1.21)	4.04E−03	1.7E−02	3700/4700	1.03 (0.94–1.12)	0.45	–
East Asian	23,406/25,654	1.11 (1.05–1.17)	1.54E−04	4E−03	11,475/12,827	1.2 (1.02–1.3)	0.023	0.0001	11,475/12,827	1.17 (1.08–1.26)	5.62E−05	1E−03	11,475/12,827	1.06 (1.01–1.1)	0.011	0.07
European	19,936/29,268	1.12 (1.04–1.2)	1.50E−03	4E−02	9968/14,634	1.14 (1.05–1.22)	0.00054	0.006	9968/14,634	1.13 (1.02–1.25)	0.013	0.4	10,390/14,812	0.98 (0.93–1.04)	0.62	–
Greater Middle Eastern	7574/7382	1.2 (1.02–1.41)	2.13E−02	5E−01	3747/3671	1.14(0.97–1.34)	0.1	–	3787/3691	1.23(1.009–1.5)	0.04	0.6	3787/3691	1.14 (0.93–1.39)	0.19	–
South Asian	2608/2176	1.04 (0.8–1.3)	6.80E−01	–	1149/988	1.34 (0.58–2.9)	0.48	–	1149/988	0.87 (0.73–1.04)	0.146	–	1149/988	0.71 (0.45–1.13)	0.15	–

The FPRP analysis was done for the significant associations. The FPRP level of noteworthiness is 0.2

*OR* odds ratio, *CI* confidence interval, *FPRP* false-positive report probability.

##### rs5210

For the rs5210 polymorphism, we found that in nine eligible studies, two studies were not followed the Hardy–Weinberg equilibrium and were removed from the analysis. Therefore, a meta-analysis was performed on seven included studies containing 4847 cases and 4642 controls from Eastern and Southern Asian populations (Table 1). As the heterogeneity was significant across the included studies with all genetic models (I^2^ = 47–83%, p-value < 0.05), we applied the random-effects model for the meta-analysis. According to our results, rs5210 appears not to be associated with T2D risk under any genetic models (Table 2). The Forest plot is also available in Supplementary Fig. S1.

##### rs5215

For the rs5215 polymorphism, we recognized ten eligible studies, one of which deviated from the Hardy–Weinberg equilibrium. Hence, the meta-analysis was conducted on the nine included studies that covered 4487 cases and 4943 controls, as presented in Table 1. Considering the significant heterogeneity across the studies under all four genetic models (I^2^ = 92–98%, p-value < 0.05), the meta-analysis was performed using the random-effects model. As Table 2 indicates, rs5215 can significantly increase the risk of T2D development only in the recessive model in the general population (combined OR = 2.07, CI 1.17–3.69, p-value = 0.013). However, the computed FPRP value (0.2) suggested that the observed association is moderate (Table 2). The forest plot can be also found in Supplementary Fig. S2.

#### Sensitivity analysis

The sensitivity analysis was conducted to assess each study's weight on the combined OR, via sequential elimination of each study under four genetic models (allelic, recessive, dominant, and over-dominant). The meta-analysis results of all three polymorphisms remained constant (Supplementary Fig. S3), suggesting that none of the included studies could significantly influence the combined OR and that our findings are stable and robust.

#### Publication bias

We applied the Egger’s test and funnel plot to evaluate the publication bias across the studies. Although we observed no publication bias for rs5210 and rs5215 in all four genetic models, it was detected for rs5219 under allele contrast, recessive, and dominant models in the general population as represented by p-value < 0.05 for the Egger’s test (Table 2). Therefore, Duval and Tweedie test was applied to correct the bias and calculate the adjusted effect size. As a result, the adjusted odds ratio with all genetic models has only slightly changed (Supplementary Fig. S4). The funnel plot for rs5210 and rs5215 with all tested genetic models was also available in Supplementary Fig. S5.

#### Meta-regression analysis

Since we have observed the significant heterogeneity among the included studies for rs5219, the meta-regression analysis was conducted to find the probable source of heterogeneity. According to our results, the pooled effect size was not significantly associated with the ethnicity, the mean age of case, the mean age of control, the mean BMI of case, and the mean BMI of control (Table 4). However, interestingly, we detected a significant association of combined effect size with the sample size of both case and control groups. As Table 4 demonstrates, the mean effect size in studies with more than 1000 cases is 0.115, 0.142, and 0.147 lower than in studies with less than 1000 cases, which explained 23%, 26%, and 8% of the observed heterogeneity in the allele contrast, dominant, and recessive genetic models, respectively. Similarly, the mean effect size of studies with more than 1000 control individuals was 0.1, 0.117, and 0.135 lower than studies with less than 1000 controls in the allele contrast, dominant, and recessive models, respectively. It accounts for 16% of the detected heterogeneity under allele contrast and dominant models but only 5% in the recessive model (Table 4). Here, we did not consider the over-dominant model as the rs5219 association meta-analysis did not produce significant results under this model in the overall analysis.

**Table 4:** Meta-regression results for rs5219.

Variable	Allele contrast		Recessive		Dominant	
	Effect size	p-value	Effect size	p-value	Effect size	p-value
Mean BMI case	− 0.0096 (− 0.03 to 0.013)	0.42	− 0.003 (− 0.04 to 0.03)	0.8	− 0.015 (− 0.04 to 0.016)	0.33
Mean age case	0.0036 (− 0.006 to 0.013)	0.46	0.0029 (− 0.017 to 0.023)	0.78	0.005 (− 0.009 to 0.018)	0.48
Sample size case	− 0.1145 (− 0.19 to − 0.03)	0.0035	− 0.15 (− 0.28 to − 0.007)	0.038	− 0.14 (− 0.2 to − 0.03)	0.0064
Mean BMI control	0.0041 (− 0.025 to 0.034)	0.78	0.008 (− 0.04 to 0.06)	0.75	0.002 (− 0.03 to 0.043)	0.9
Mean age control	0.0025 (− 0.003 to 0.0083)	0.4	− 0.001 (− 0.013 to 0.011)	0.86	0.0048 (− 0.003 to 0.012)	0.22
Sample size control	− 0.1 (− 0.17 to − 0.023)	0.01	− 0.14 (− 0.2 to 0.003)	0.05	− 0.12 (− 0.21 to − 0.014)	0.025

BMI and age are quantitative traits. Sample size was considered a binary trait. Studies with fewer than 1000 samples and those with more than or equal to 1000 samples were categorized as small and large sample sizes, respectively.

## Discussion

The sufficient sample size and the consequent statistical power are important prerequisites for acquiring reliable genetic association results and decoding the genetic architecture of complex diseases, such as type 2 diabetes. To achieve this goal, combining various genetic association studies in the form of meta-analysis is an appropriate approach. Here, we examined the association of common variants of *KCNJ11* with type 2 diabetes incidence in the Iranian cohort, followed by carrying out the most comprehensive meta-analysis to assess the potential role of *KCNJ11* polymorphisms in T2D susceptibility. Since rs5210, rs5215, and rs5219, had adequate published association studies with T2D worldwide, the current study focused on these *KCNJ11* polymorphisms. Previous studies reported that the association between rs5219 and increased type 2 diabetes risk could be partly explained by a reduced insulin secretion resulting from rs5219^23,24^. However, in the present study, we figured out that the insulin secretion level is similar in non-diabetic participants with different rs5219 genotypes, which is in line with the lack of association of this polymorphism with type 2 diabetes development across Iranian adults.

Based on our meta-analysis, the role of rs5219 is varied among different ethnicities. It can raise the risk of T2D development in all tested ethnicities, except for South Asia, where this polymorphism is not apparently involved in T2D incidence. Based on FPRP value, the detected genetic association can be categorized as strong (FPRP < 0.05), moderate (0.05 ≤ FPRP ≤ 0.2), or weak (FPRP > 0.2)^26^. Here, the FPRP analysis suggested the weak association of this polymorphism with T2D in the Great Middle East population, which is in agreement with our findings in the Iranian population. However, we did not observe any association of rs5219 with T2D in the South Asian subgroup. Generally, the genetic association results are not consistent among different ethnicities. The Ensembl database reported the risk allele frequency (RAF) of rs5219 from 0.02 in African to 0.4 in South Asia^27^. Our findings can also be attributed to the distinct linkage disequilibrium (LD) structure among rs5219 and adjacent probable causal variants in different ethnic groups. Both RAF and LD patterns have become different across ethnicities during evolutionary processes^28^. Additionally, differences in non-genetic factors, including the disease age of onset and lifestyle, can lead to the discrepancy. Between-study heterogeneity is the common issue in the meta-analysis, which can refer to differences in age, BMI, and genetic background of participants in each study, over-estimation of genetic effect, differences in LD structure among the population, and sample size used for each study^27,28^. For instance, Peng et al. suggested that differences in mean BMI in the control group is one of the heterogeneity sources of association meta-analysis of rs9939609 FTO with obesity risk^28^. Despite detecting significant heterogeneity for all three polymorphisms in the present study, we could perform the meta-regression analysis for rs5219 but not for rs510 and rs5215 due to insufficient information. Among the tested variables, the sample size of both case and control groups had significantly influenced the combined effect size under all tested genetic models. We indicated that studies more than 1000 cases or 1000 controls have been generated a lower pooled odds ratio as compared to studies with less than 1000 subjects. It is highlighted the importance of adequate case and control sample size in genetic association studies to achieve the accurate results, especially when the expected effect size is low. Similarly, the previous studies recommended the higher samples size for conducting the genetic association analysis with rare variants or common variants with low effect size^29,30^. In a prior meta-analysis of rs5219^33^, the authors hypothesized that heterogeneity might have resulted from the younger mean age of the control group as T2D is a disease with a relatively late-onset. But our meta-regression results demonstrated that the mean age and the mean BMI of case and control groups were not associated with the pooled effect size and could not be the source of heterogeneity. rs5210 can positively affect T2D susceptibility, which is in line with a previous meta-analysis conducted on only two studies^34^. Although we performed an extensive meta-analysis association of this polymorphism with nine eligible studies for the first time, all of them belonged to East and South Asia, which may introduce a potential bias to the result. Consequently, further studies from diverse ethnicities are required to generalize the findings.

From the potential clinical usage perspective, molecular biomarkers like SNPs have broad clinical applications in disease screening, early diagnosis, and treatment efficiency, which are the primary goals of precision medicine. Hence, genetic association studies are critical for recognizing risk variants associated with complex diseases, including type 2 diabetes. The identified risk variants can be utilized to detect genetically susceptible individuals at a very young age before appearing T2D symptoms. However, on the one hand, the associated genetic variants display diverse impacts among different ethnic groups. On the other hand, single genetic association studies are usually not robust enough to detect effective variants due to the small sample size. But association meta-analysis studies can overcome this issue by combining all eligible studies, generating more precise results. Alongside other risk SNPs, the present meta-analysis results could guide us to consider the *KCNJ11* rs5219 and rs5215, but not rs5210, in designing an efficient SNP-based panel for potential clinical usage in the near future. Consequently, the individuals with the highest genetic risk can be detected early and subjected to lifestyle improvement and other interventions to prevent or delay T2D. Eventually, it will reduce the type 2 diabetes rate and its complications, gradually increase life quality and decrease the economic cost of the disease.

There are some strengths and weaknesses in our study that merit discussion. Firstly, compared with previously published meta-analyses^31–33^, the present study comprised up-to-date and more literature reviews with the homogenous study design (case–control or nested case–control). Secondly, unlike the previous meta-analyses, we classified different ethnicities according to genetic information, not geographical area, for performing subgroup analysis based on ethnicity. Thirdly, we made an effort to find the potential source of between-study heterogeneity by doing an extensive meta-regression analysis with all genetic models and obtaining the consistent results. However, type 2 diabetes has a complicated genetic architecture that numerous genetic variants shape it, while we considered only essential variants of the *KCNJ11* gene in the current study. Additionally, due to the lack of accessibility to the appropriate covariates, such as age, gender, and BMI, in the various studies, we could not adjust the pooled genetic effect size for those variables. Hence, the meta-analysis results should be interpreted with caution. Publication bias is a usual event in any meta-analysis study. Here, we found it only for rs5219 and adjusted it using the trim and fill method, however, the lack of publication bias cannot be a certainty sign of meta-analysis since the studies with significant association results usually have more chance of being published than studies with insignificant association results. Therefore, we should note that even if the publication bias is not detected in the meta-analysis, it cannot be completely ruled out.

To conclude, the current study can be considered the most comprehensive association meta-analysis of *KCNJ11* polymorphisms with type 2 diabetes incidence, which can expand our knowledge about the role of this gene in T2D incidence. However, more investigations are required to dissect the impact of *KCNJ11* polymorphisms on insulin secretion levels. Furthermore, we recommend that researchers consider collecting studies with similar sample sizes to reduce the between-study heterogeneity in the meta-analysis studies.

## Methods

### Nested case–control study

#### Subjects and measurements

In the current study, Iranian subjects were opted from an ongoing TCGS project that is a branch of a Tehran Lipid and Glucose Study (TLGS) longitudinal project, in which participants have been genotyped and followed up for cardio-metabolic risk factors every three years since 1999 (1999–2017)^36^. The ethics committee approved all procedures performed in this study on human subject research at Research Institute for Endocrine Sciences, Shahid Beheshti University of Medical Sciences (code of “IR.SBMU.ENDOCRINE.REC.1395.366″), which were following the 1964 Helsinki Declaration and its later amendments or comparable ethical standards. At each visit, written consent was obtained from each subject and referred to trained physicians and laboratories for clinical examinations and blood sampling. Considering the attendance of individuals from the main Iranian ethnic groups living in Iran, including Persians, Azeris, Kurds, Lors, Arabs, Baluchs, Turkmans, Mazanis, and Gilaks at the TCGS cohort, this cohort could represent the Iranian population. In summary, weight and height were recorded using the standard protocols. Body mass index (BMI) was calculated as weight in kilograms divided by height in square meters. Fasting plasma glucose (FPG), 2-h plasma glucose, and fasting insulin concentration was measured using the standard protocols^37^. Type 2 diabetes was diagnosed based on the fasting plasma glucose ≥ 126 mg/dL or 2-h plasma glucose ≥ 200 mg/dL during an oral glucose tolerance test or usage of anti-diabetic drugs. The first occurrence of type 2 diabetes in individuals during the follow-up period was considered as diabetic condition^38^.

#### Genotyping and genetic association analysis

Genomic DNA of TCGS participants was extracted from the white blood cells via an alkaline boiling method, and its quantity and quality were evaluated by electrophoresis and spectrophotometry. DNA samples of 13,693 TCGS participants were genotyped using Illumina Human OmniExpress-24-v1-0 bead chip at the deCODE genetics company (Iceland) according to manufacturer's specifications (Illumina Inc., San Diego, CA, USA)^39^. Three common *KCNJ11* polymorphisms (rs5210, rs5215, and rs5219) were chosen for this study. Before running the analysis and for making sure that our sample size is adequate, we estimated the statistical power using GAS power calculator^40^. For power calculation, we considered the given sample size, disease prevalence of 11.4% in Iran^41^, the minor allele frequency of 0.34, the average odds ratio of 1.2 for three SNPs of interest obtained from previous studies, and the significance level of 0.01.

After relevant quality control of genotyped SNPs and individuals using the PLINK program (V. 1.9), we investigated their association with type 2 diabetes incidence through the logistic regression analysis under six genetic models (allele contrast, recessive, dominant, over-dominant, codominant heterozygous, and codominant homozygous) adjusted for the BMI, sex, and age. The false discovery rate (FDR) at the 5% significance level was considered for correcting multiple testing. For this study, we considered 2991 (1326 diabetic and 1593 non-diabetic) unrelated adults (≥ 20 years old). Participants less than 20 years old, related adults, and pre-diabetic adults were excluded from the analysis.

#### Pancreatic β-cell function measurement

The β-cell function was estimated using the Homeostatic Model Assessment of β-cell function (HOMA-β) formula: HOMA-β = 20 × fasting insulin (μU/mL)/fasting glucose (mmol/L) − 3.5)^42^. We used an analysis of variance (ANOVA) test to compare the β-cell function mean among non-diabetic participants with various rs5219 genotypes possessing zero, one, and two risk alleles. p-value < 0.05 was considered significant.

### Meta-analysis

The current meta-analysis study was done based on the criteria of the Preferred Reporting Items for Systematic Reviews and Meta-analyses (PRISMA)^43^. A PRISMA checklist is provided as supplementary file S2. The present study was not registered. The review protocol can be accessed on reasonable request.

#### Literature search and inclusion criteria

Manuscripts that investigated the genetic association between *KCNJ11* rs5210, res4215, and rs5219 with type 2 diabetes incidence were selected from PubMed, Scopus, and Google Scholar, and ProQuest based on publication date from 1990 to Jan. 2022. We used the following key words: “KCNJ11”, “Kir 2.6 channel”, “potassium inwardly-rectifying channel, subfamily J, member 11 protein, human”, “Potassium Voltage-Gated Channel Subfamily J Member 11”, and “Inwardly-Rectifying Potassium Channel Subfamily J Member 11”, T2D”, “type 2 diabetes”, “Non-Insulin-Dependent Diabetes Mellitus”, “Ketosis-Resistant Diabetes Mellitus”, “polymorphism”, “SNP”, “rs5210”, “rs5215”, and “rs5219”. The search was restricted to human studies published in the English language.

In the current meta-analysis, we included the eligible studies with the following criteria: (1) published in peer-reviewed journals with available full text. (2) Designed as the case–control, nested case–control, and genome-wide association study. (3) Provided distributions of genotypes or alleles in case and control groups for calculating the odds ratio (OR) with the corresponding 95% confidence intervals (CI) and p-value. (4) Diagnosed T2D based on American Diabetes Association (ADA). In contrast, we excluded (1) case-only studies, (2) review, meta-analysis, editorial, and conference abstracts, (3) family-based association studies, and (4) studies without sufficient genotype frequency data that OR could not be calculated.

#### Data extraction and quality assessment

Two reviewers independently evaluated studies for relevance in a standardized manner. In case of disagreement, it was resolved by discussion and adjudication with the third reviewer. After removing duplicated records, the following information was extracted: first author’s name and publication year, participants’ ethnicity, the distribution of polymorphisms of interest in cases and controls, genotyping method, mean age, and BMI of cases and controls, and sample size of cases and controls. Newcastle–Ottawa scale (NOS) was applied to assess the methodological quality of eligible studies as previously described^44^. The score on this scale ranged between zero and nine; studies with the at least score of seven were presumed to be a high-quality study.

#### Statistical analysis

For the present meta-analysis, the Hardy–Weinberg Equilibrium (HWE) was assessed in the control group of each study. The studies that have not obeyed the HWE rule (p-value > 0.05) were removed from further analysis. The effect of polymorphisms of interest (rs5210, rs5215, and rs5219) on type 2 diabetes development was evaluated under four genetic models, allele contrast, recessive, dominant, and over-dominant. Results were presented as the pooled odds ratio (OR) with 95% confidence interval (CI), and the related p-value. To detect the possible heterogeneity among the studies, we utilized the Cochrane Q-test and I^2^ statistics. The random-effect model was applied if there was a significant heterogeneity (p-value < 0.05, I^2^ > 50%). Otherwise, the fixed-effect model was used. Furthermore, we computed the false positive report probability (FPRP) to detect the potential false-positive results among the significant association meta-analysis findings. As proposed by Wacholder et al., the prior probability of 0.05 and the FPRP cutoff value of 0.2 were considered for this analysis^45^. To survey the potential source of heterogeneity, we conducted meta-regression analysis using the ethnicity, mean age of control group, mean age of case group, mean BMI of case group, mean BMI of the control group, and sample size of case and control groups (N ≥ 1000 or < 1000). BMI and age were considered quantitative traits while sample size was considered a binary trait. Studies with fewer than 1000 samples and those with more than or equal to 1000 samples were categorized as small and large sample sizes, respectively, studies with small sample size were considered as reference for doing the regression analysis. Ethnic groups were specified as American, African, Central Asian, European, Eastern Asian, Greater Middle Eastern, Southern Asian, and others based on Morales et al.'s genetic ancestry classifications^46^. Additionally, we appraised the robustness of our meta-analysis results by leave-one-out method in the sensitivity analysis via consecutive excluding only one study each time to assess the possible impact of a single study on the final result (pooled OR). Besides, we examined the potential publication bias using Egger’s test and visualized it by drawing a funnel plot. If the publication bias was detected, a Duval and Tweedie trim-and-fill method was applied to compute the adjusted effect size^47^. All analysis was performed using STATA (version 11). We used the MetaGenyo tool for presenting the high-resolution figures^48^.

## Supplementary Information

Supplementary Information.

## Data Availability

The data analyzed during the current study for the Iranian population are available from the corresponding author on reasonable request. Other data used for meta-analysis is available at Table 1.
