# Supplementary material for: Risk of type 2 diabetes and KCNJ11 gene polymorphisms: a nested case–control study and meta-analysis
Source: Sci Rep. 2022 Dec 1;12:20709. doi: 10.1038/s41598-022-24931-x (PMC9715540; doi:10.1038/s41598-022-24931-x)

**Supplementary Tables and Figures for:**

Risk of Type 2 Diabetes and *KCNJ11* Gene Polymorphisms: A Nested Case-control Study and Meta‑analysis

**Supplementary Table 1.** Baseline characteristics of the TCGS cohort (1999–2017) participants used in the present study

| Variables | Diabetic | Non-diabetic |
| --- | --- | --- |
| Sex (Male/Female, number) | 575/750 | 610/983 |
| Age (mean ± SD) | 50.41 ± 12 | 37.53 ± 13 |
| Body mass index (mean ± SD) | 28.98 ± 4.6 | 25.58 ± 4.7 |
| Fasting plasma glucose (mean ± SD) | 124.4 ± 51.5 | 85 ± 6.57 |
| 2-h plasma glucose (mean ± SD) | 171.67 ± 84.2 | 93 ± 19.4 |

**Supplementary Table 2** Association of *KCNJ11* polymorphisms with type 2 diabetes development among Iranian adults. Models were adjusted for age, sex, and BMI.

|  |  | Additive model | | Dominant model | | Recessive model | | | Over-dominant model | |
| --- | --- | --- | --- | --- | --- | --- | --- | --- | --- | --- |
| SNP and Effect allele | **EAF** | **OR (95% CI)** | **FDR** | **OR (95% CI)** | **FDR** | | **OR (95% CI)** | **FDR** | **OR (95% CI)** | **FDR** |
| rs5210-A | 0.281 | 0.92 (0.8-1.05) | 0.72 | 0.94 (0.8-1.13) | 0.89 | | 0.7 (0.57-1.06) | 0.64 | 0.85 (0.6-1.07) | 0.88 |
| rs5215-T | 0.359 | 1.023 (0.9-1.16) | 0.7 | 1.008 (0.8-1.2) | 0.9 | | 1.08 (0.83-1.4) | 0.9 | 1.05 (0.72-1.2) | 0.93 |
| rs5219-C | 0.36 | 1.03 (0.9-1.17) | 0.7 | 1.01 (0.85-1.2) | 0.92 | | 1.09 (0.84-1.43) | 0.92 | 0.96 (0.83-1.12) | 0.8 |

EAF: Effect allele frequency, OR: odds ratio, CI: confidence interval, FDR: False discovery rate

**Supplementary Table 3.** Comparison of β-cell function (log-transformed) among non-diabetic participants with different rs5219 genotypes

| Genotype | Sample size | mean ± SD | p-value |
| --- | --- | --- | --- |
| CC | 334 | 3.22 ± 0.55 | 0.69 |
| CT | 377 | 3.2 ± 0.6 |  |
| TT | 97 | 3.17 ± 0.54 |  |

**Supplementary Fig.1** Forest plot for the association between rs5210 and type 2 diabetes risk under different genetic models. Diamond shows the pooled odds ratio size and its 95% CI

**
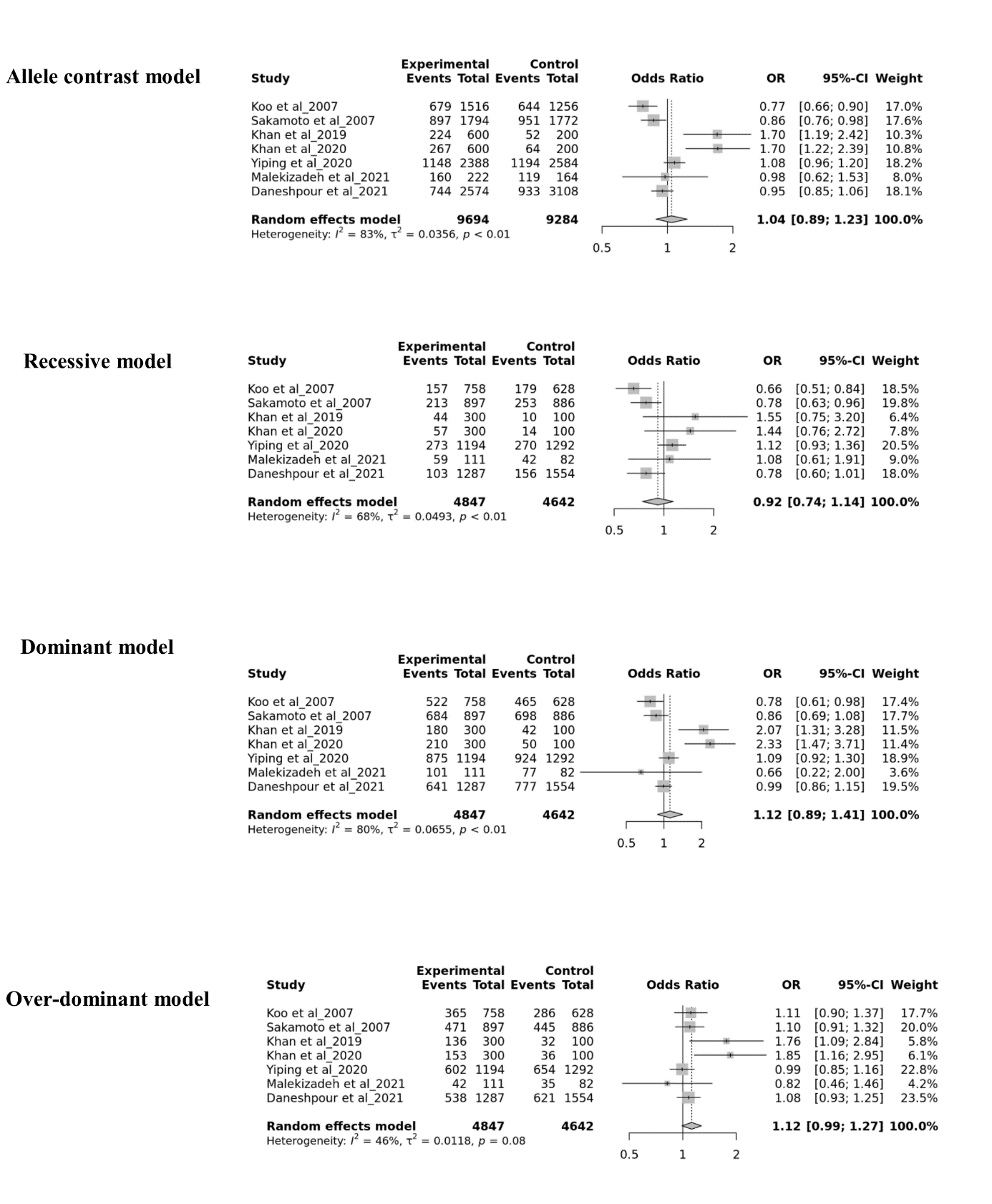
**

**Supplementary Fig.2** Forest plot for the association between rs5215 and type 2 diabetes risk under different genetic models. Diamond shows the pooled odds ratio size and its 95% CI

**
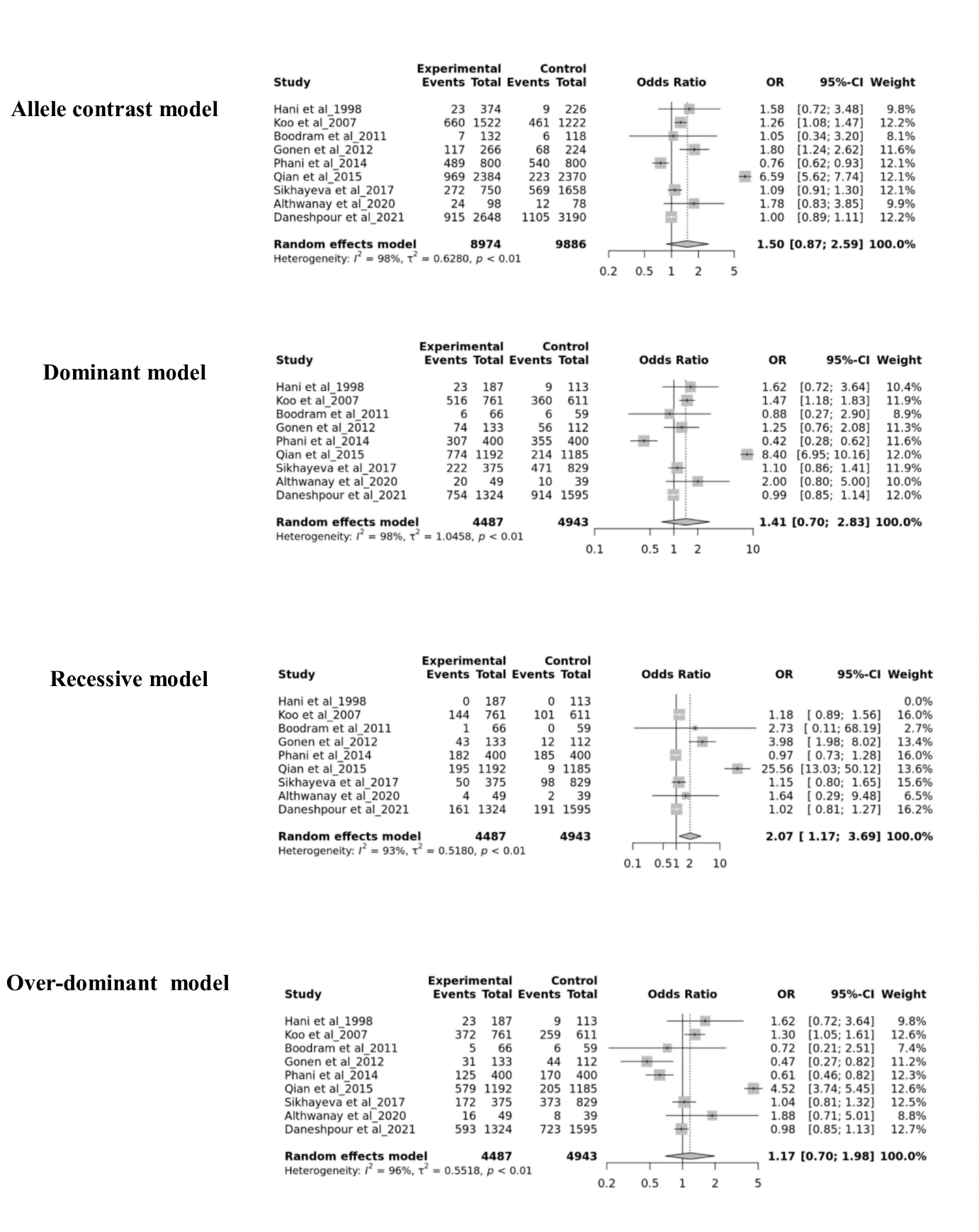
**

**Supplementary Fig. 3** Sensitivity analysis of meta-analysis association of rs5210, rs5215, and rs5219 with type 2 diabetes development under different genetic models, allele contrast (A), recessive (B), dominant (C), and over-dominant (D) models.


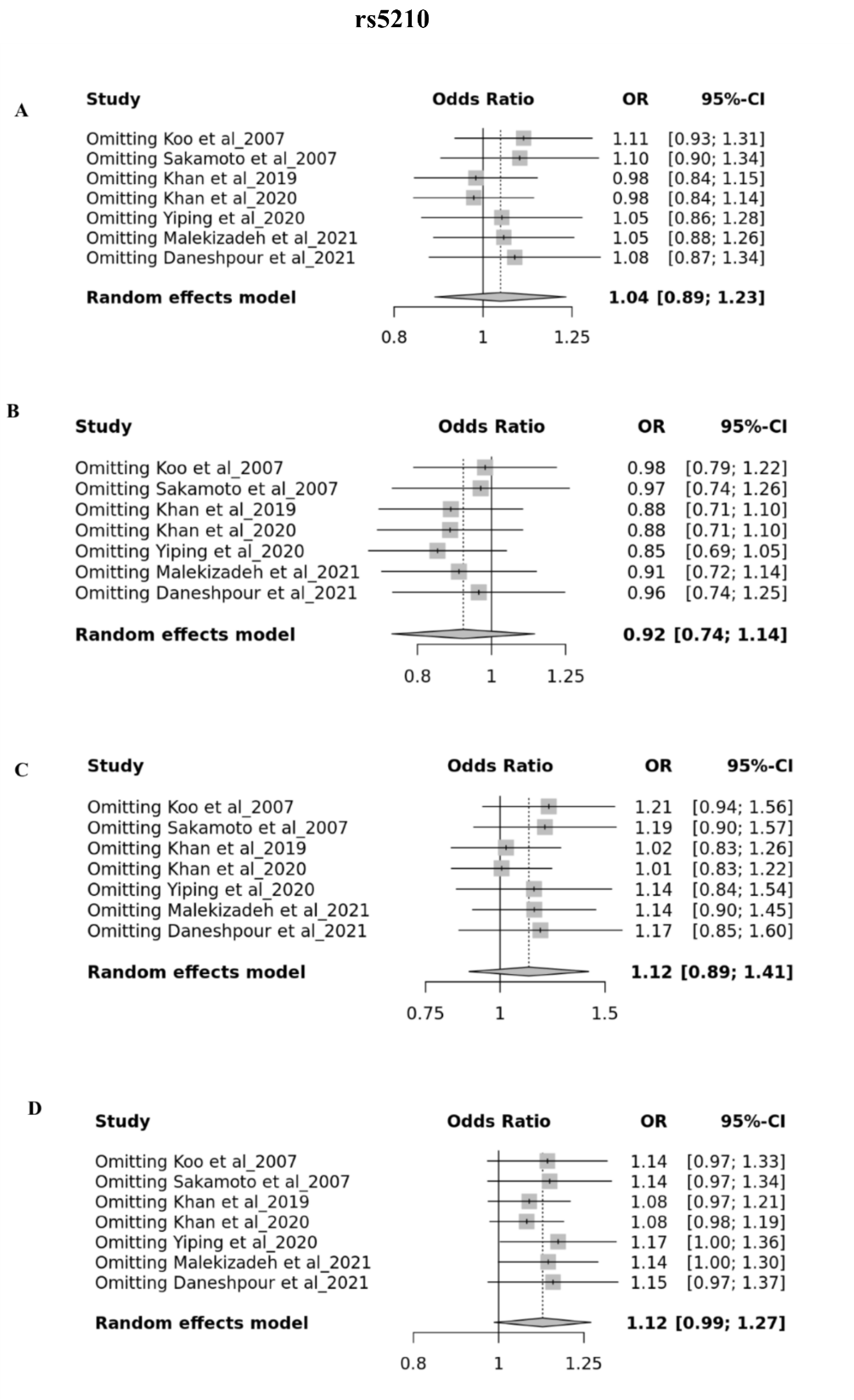


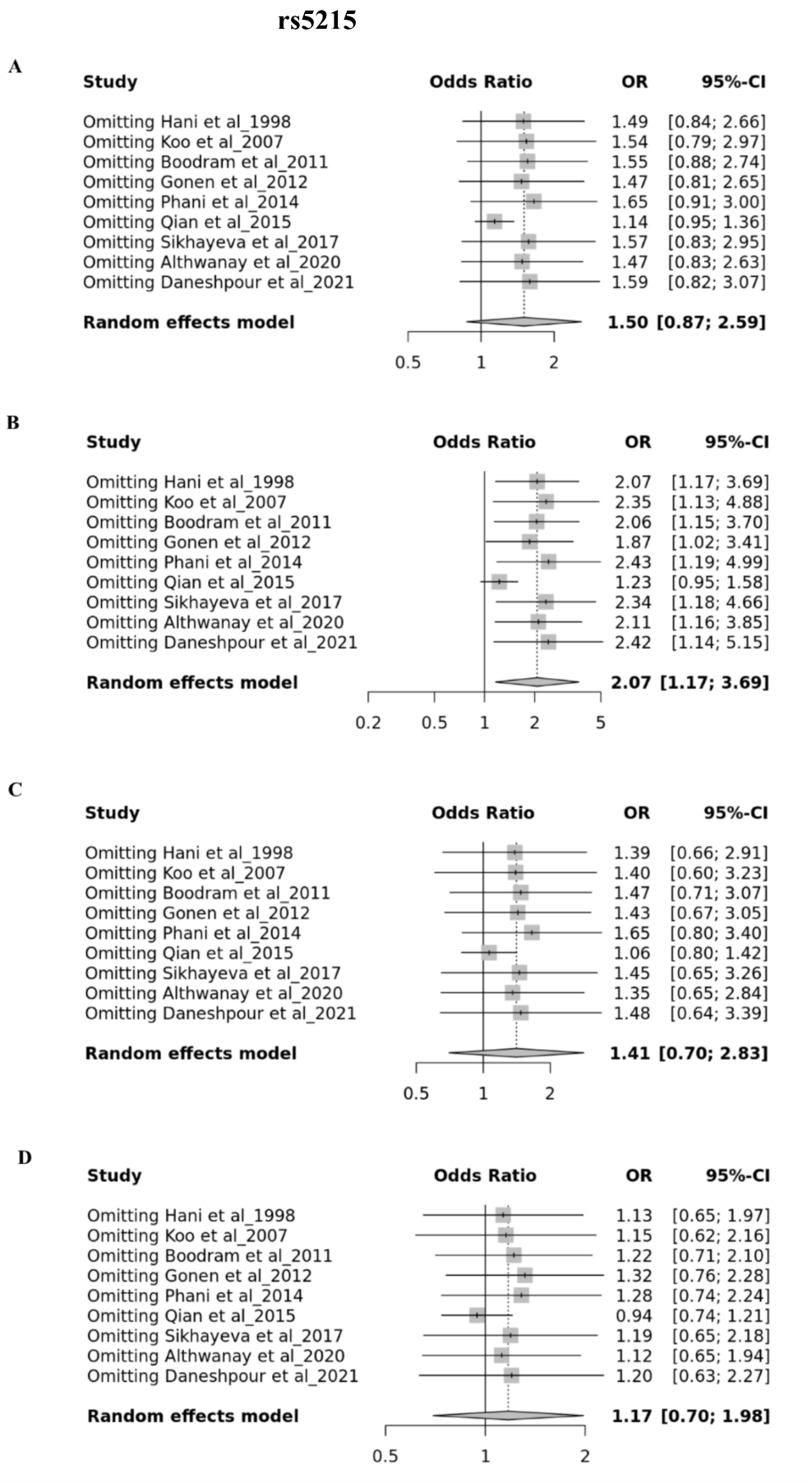


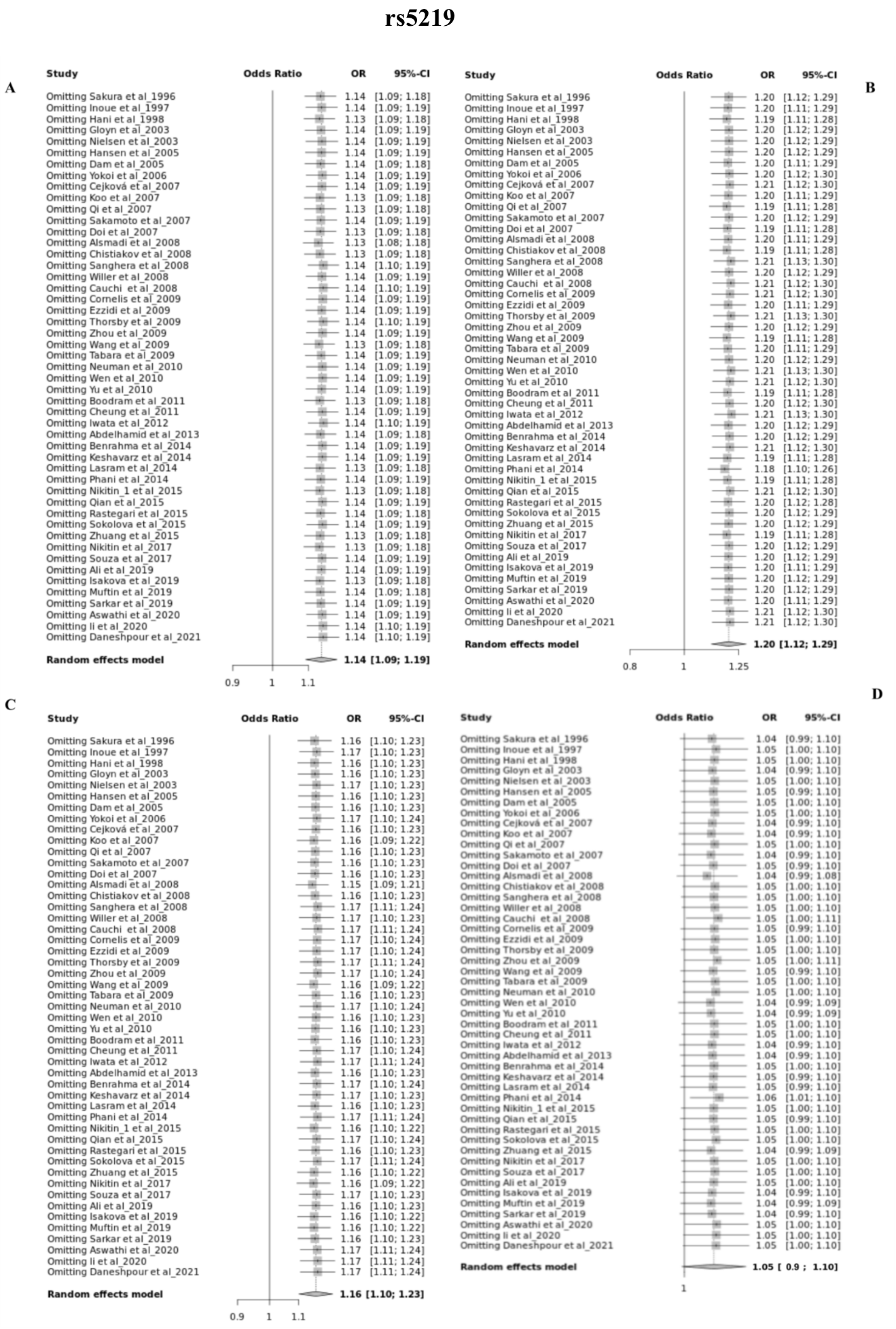


**Supplementary Fig. 4** Funnel plot of the meta-analysis association for rs5219 under allele contrast (A), recessive (B), and over-dominant (C) models. Each point represents the log odds ratio and the standard error for a single study. The empty circles are observed studies and the filled circles are imputed studies for correcting the publication bias.

**
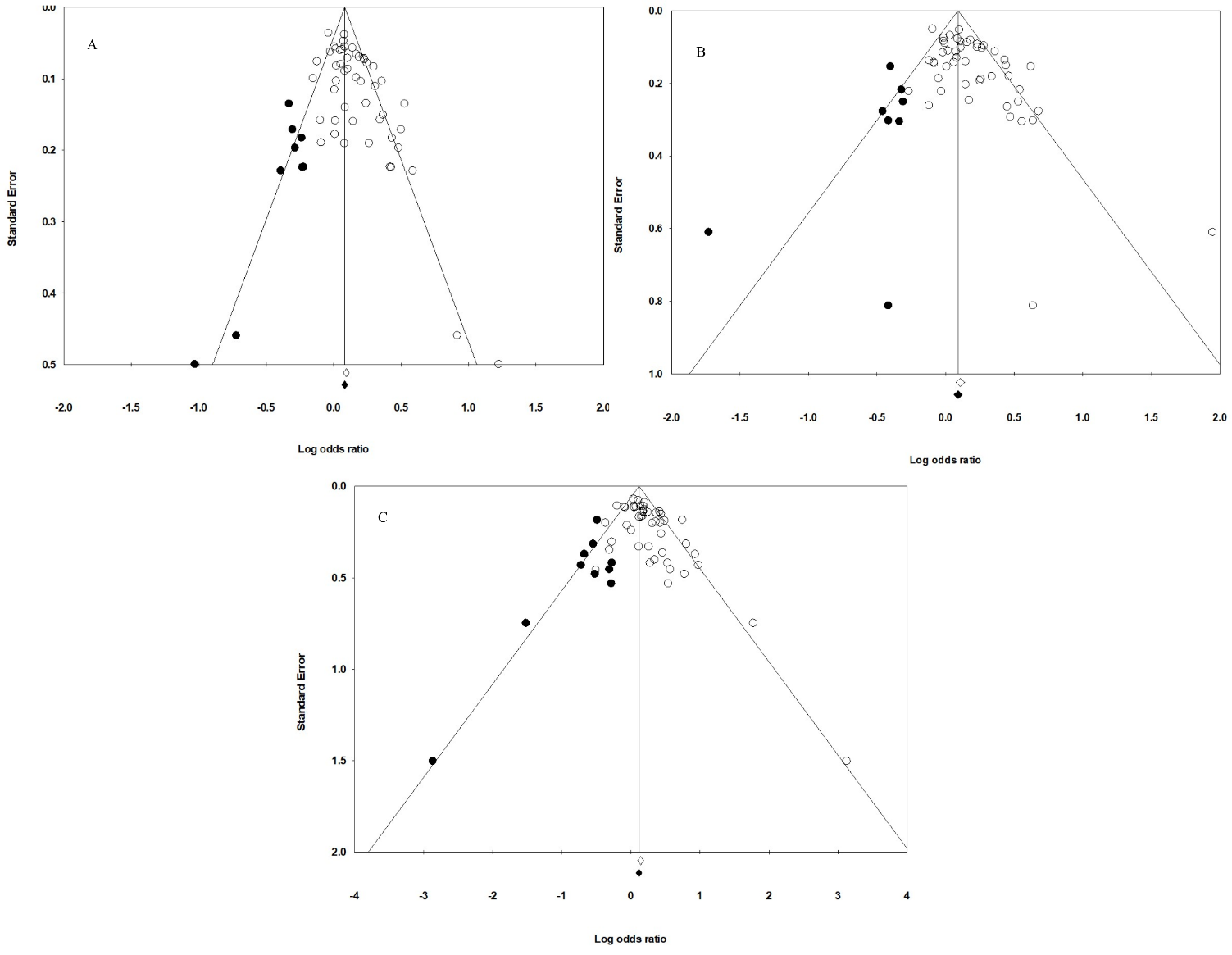
**

**Supplementary Fig. 5** Funnel plot of the meta-analysis association for rs5210 and rs5215 under different genetic models, allele contrast (A), recessive (B), dominant (C), and over-dominant (D) models.


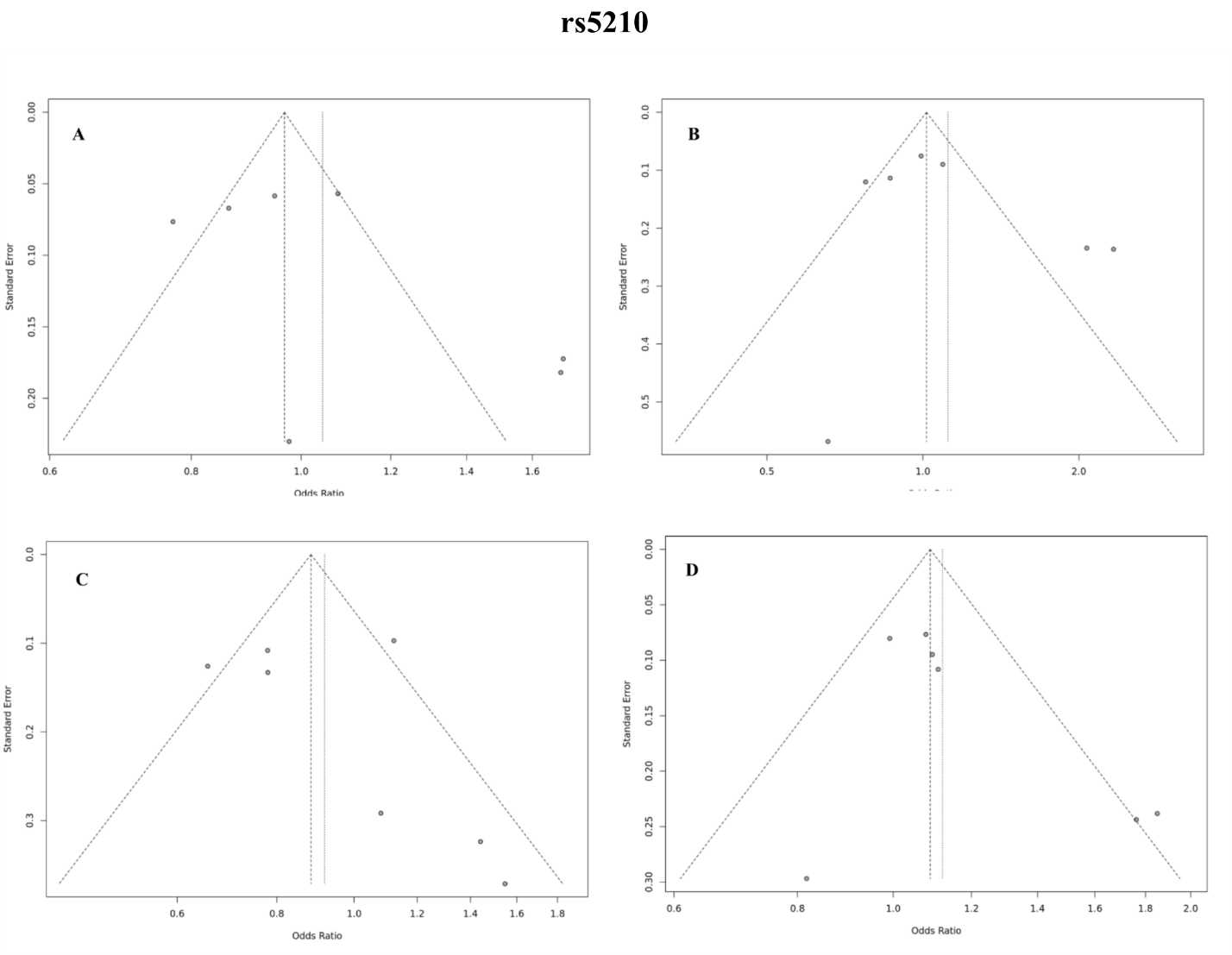


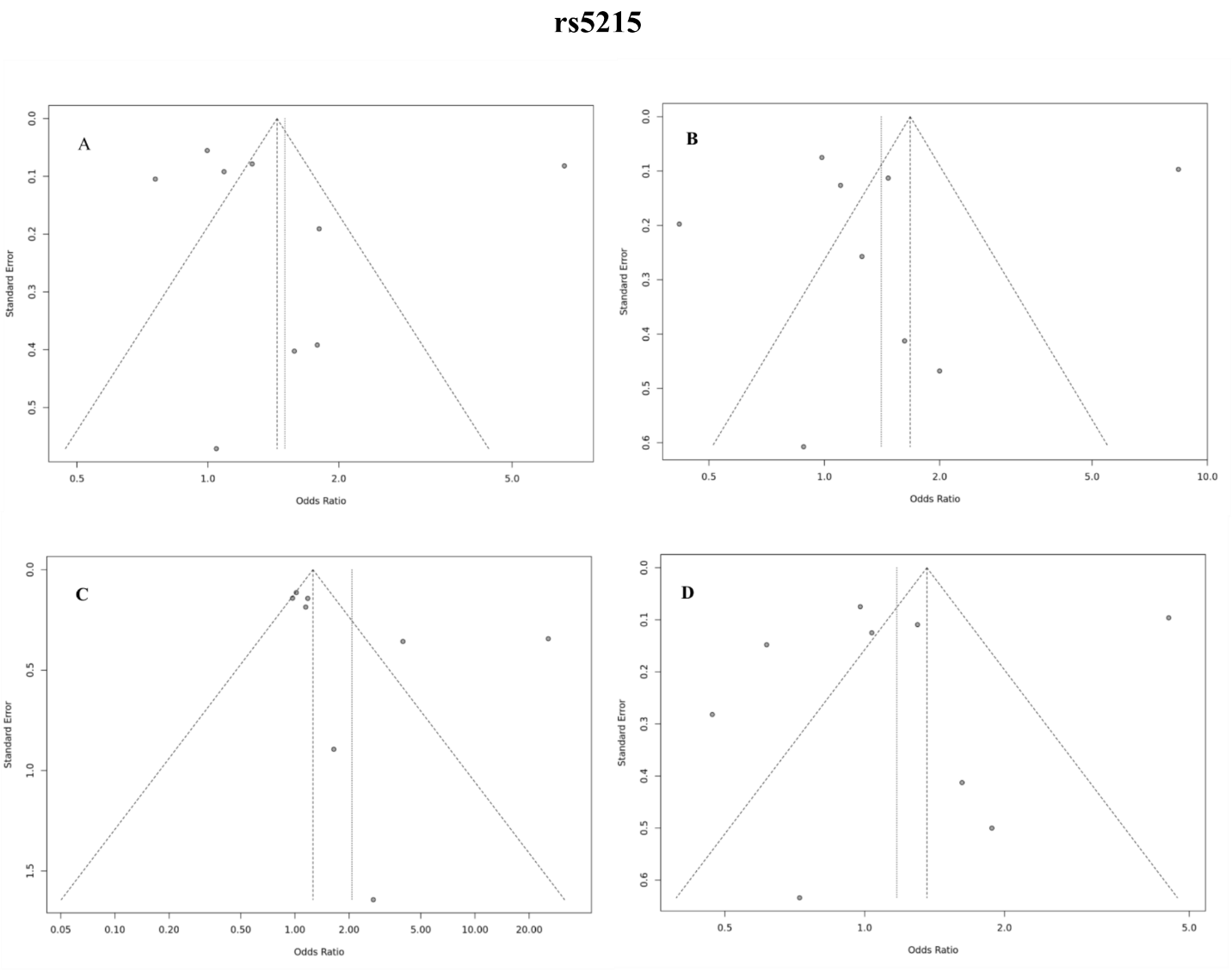

Supplement: Supplementary file 1 — Supplementary Information. [file 41598_2022_24931_MOESM1_ESM.docx]
